# Detection of *bla*_IMP4_ and *bla*_NDM1_ harboring *Klebsiella pneumoniae* isolates in a university hospital in Malaysia

**DOI:** 10.3402/ehtj.v8.26011

**Published:** 2015-03-10

**Authors:** Nurul Izzati Hamzan, Chan Yean Yean, Rosliza Abdul Rahman, Habsah Hasan, Zaidah Abdul Rahman

**Affiliations:** Department of Medical Microbiology & Parasitology, School of Medical Sciences, Universiti Sains Malaysia, Kubang Kerian, Kelantan, Malaysia

**Keywords:** carbapenem-resistant *Enterobacteriaceae*, carbapenemase, *Klebsiella pneumoniae*, Modified Hodge test

## Abstract

**Background:**

Antibiotic resistance among *Enterobacteriaceae* posts a great challenge to the health care service. The emergence of carbapenem-resistant *Klebsiella pneumoniae* (CRKP) is attracting significant attention due to its rapid and global dissemination. The infection is associated with significant morbidity and mortality, thus creating challenges for infection control and managing teams to curb the infection. In Southeast Asia, there have been limited reports and subsequent research regarding CRKP infections. Thus, the study was conducted to characterize CRKP that has been isolated in our setting.

**Methods:**

A total of 321 *K. pneumoniae* were included in the study. Each isolate went through an identification process using an automated identification system. Phenotypic characterization was determined using disk diffusion, modified Hodge test, Epsilometer test, and inhibitor combined disk test. Further detection of carbapenemase genes was carried out using polymerase chain reaction and confirmed by gene sequence analysis.

**Results:**

All together, 13 isolates (4.05%) were CRKP and the majority of them were resistant to tested antibiotics except colistin and tigercycline. Among seven different carbapenemase genes studied (*bla*_KPC_, *bla*_IMP_, *bla*_SME_, *bla*_NDM_, *bla*_IMI_, *bla*_VIM_, and *bla*_OXA_), only two, *bla*_IMP4_ (1.87%) and *bla*_NDM1_ (2.18%), were detected in our setting.

**Conclusion:**

Evidence suggests that the prevalence of CRKP in our setting is low, and knowledge of Carbapenem-resistant *Enterobacteriaceae* and CRKP has improved and become available among clinicians.

The overuse and misuse of antibiotics have led to the emergence of resistance in bacteria. Problems related to multidrug-resistant (MDR) organisms or superbugs are worrisome these days as they are becoming increasingly serious (1). One of the mechanisms of resistance in *Enterobacteriaceae* is caused by hydrolyzing enzymes, the most important being carbapenem-hydrolyzing enzyme (2). Carbapenem-resistant *Enterobacteriaceae* (CRE) is emerging, and carbapenem-resistant *Klebsiella pneumoniae* (CRKP) is the most common CRE detected.

Since its first detection in 1996 in North Carolina, CRKP has been reported worldwide, including in Asia (3–6). In Southeast Asia, a few cases of infection associated with CRKP were recently reported in Singapore (7). Therefore, there is evidence that CRKP isolates have undergone extensive dissemination in many countries and continue to spread in new geographical locations. It is a matter of great concern that the prevalence of these pathogens has continued to rise worldwide (8).

With the emergence of CRKP, clinicians are left with very limited antibiotic options to treat infections, because CRKP is completely resistant to all antibiotics, including carbapenem (9). Further complicating the matter is the fact that infections caused by CRKP are associated with increased mortality, length of hospital stay, and cost of hospitalization (10). Therefore, early detection of CRKP and notification are crucial steps for the infection control team to implement strict infection control practice.

With reference to the Malaysian context, to date, including during the preparation of this manuscript, little published data regarding the epidemiology of CRE and CRKP was readily available. For this reason, the study attempted to determine the prevalence of the CRKP and its genotype among clinical isolates of *K. pneumoniae* in our hospital. Subsequently, knowledge of CRE and CRKP has improved and become available among clinicians.

## Methods

### Study design and setting

This cross-sectional, descriptive study was conducted between April 2010 and June 2012 in Hospital Universiti Sains Malaysia (Hospital USM), Kelantan, Malaysia. Hospital USM is a tertiary teaching hospital as well as the referral hospital for the eastern coastal region of Peninsular Malaysia. The hospital has 700 beds and 28 wards, including medical, surgical, pediatric, and orthopedic wards. It also has general, surgical, and two neonatal intensive care units (ICUs).

### Clinical isolates

The study was conducted in the Medical Microbiology & Parasitology Laboratory. *Klebsiella pneumoniae* isolated from various clinical specimens such as endotracheal aspirate, pus, sputum, urine, wound/tissue, and blood during the study period were collected and screened. Samples were processed according to standard laboratory procedures. Isolates were identified based on growth characteristics and basic biochemical testing and confirmed by the Vitek2 automated identification system, using the Vitek GNI card (bioMérieux Vitek, Durham, North Carolina, USA).

### Antibiotics susceptibility testing

Antibiotics susceptibility testing was performed for all isolates using disc diffusion method as described by Clinical and Laboratory Standard Institute and interpreted accordingly (11). The antimicrobials tested include amikacin, gentamicin, piperacillin–tazobactam, amoxicillin–clavulanate, cefepime, cefotaxime, ceftazidime, cefoperazone, cefuroxime, ciprofloxacin, trimethoprim–sulfamethoxazole, ertapenem, meropenem, and imipenem. Isolates which demonstrated a reduced susceptibility toward ertapenem, imipenem, or meropenem were further tested for minimal inhibitory concentration (MIC) by E-test method. CRKP isolates were further tested for tigercycline and colistin susceptibility.

### Phenotypic test

Modified Hodge test was performed according to the procedure recommended by Amjad et al. (12). Combined disc test with 3′ aminophenylboronic acid and ethylene-diaminetetra-acetic acid was performed as described by Hung et al. with some modifications (13). *Klebsiella pneumoniae* BAA-1705 and *K. pneumoniae* BAA-1706 were used as positive and negative controls, respectively, through out this study.

### Molecular detection and DNA sequencing

Detection of CRKP genes for CRKP isolates was conducted by conventional in-house polymerase chain reaction (PCR) using primers targeting 11 *bla*_KPC_ variants. The forward (KPC-F 5′-GCCGTCTAGTTCTGCTGTCTTG-3′) and reverse primers (KPC-R 5′-GCCCAATCCCTCGAGCGCG-3′) detecting KPC-1 to KPC-11 were designed using Vector NTI and GeneDoc software. BLAST program searches were performed using the National Center for Biotechnology Information website to check the specificity of the primer designed (4). The detection of other resistant genes, namely *bla*_SME_, *bla*_IMI_, *bla*_IMP_, *bla*_NDM_, *bla*_VIM_, and *bla*_OXA_, was done using published primers (14–18). DNA was isolated from bacterial colonies using the boiling lysis method as previously recommended (19). Internal control (*hem*M: 519-bp) was incorporated in each reaction for validation. The PCR was run using a Peltier thermal cycler (MJ Research, Watertown, Massachusetts, USA). PCR products were detected by an agarose gel electrophoresis and visualized by UV transilluminator (Alpha Innotech, San Leandro, CA, USA). PCR products were sent for DNA sequencing and compared with existing databases by multiple-sequence alignment using the BLAST program.

## Results

Altogether, 321 *K. pneumoniae* were screened in the study with 13 (4.05%) isolates showing reduced susceptibility to ertapenem, imipenem, or meropenem by disc diffusion method. Results were further confirmed using E-test. The MICs for imipenem and meropenem ranged between 0.25 and ≥32 µg/ml. The majority of patients with CRKP, 9/13 (70%), were managed in the ICU. Other patients were treated in general wards: medical (2/13), surgical (1/13), and orthopedic (1/13). Seven (54%) CRKP were isolated from endotracheal aspirate, and the rest were isolated from blood (2/13, 15%), urine (2/13, 15%), and wound swab (2/13, 15%).

All CRKP were resistant to 2nd and 3rd generation cephalosporin tested which include cefuroxime, cefepime, cefotaxime, ceftazidime, cefoperazone, trimethoprim–sulfamethoxazole, and amoxicillin–clavulanate. A few CRKP isolates were still susceptible to aminoglycosides, whereas 5/13 (38.5%) were susceptible to gentamicin, amikacin, and netilmycin. In addition, 4/13 (30.7%) of isolates were susceptible to ciprofloxacin and 4/30 (13.3%) to piperacillin–clavulanate. Eight isolates (61.5%) were resistant to all tested antibiotics except colistin and tigercycline. The results of antibiotic susceptibility testing for all CRKP isolates are shown in Table 1.

**Table 1:** Antibiotic susceptibility patterns of Carbapenem-resistant *Klebsiella pneumoniae* (CRKP)

	Antibiotic tested and zone of inhibition (mm)													
	
Isolate	ETP	MEM	IPM	SXT	AK	CN	NET	TZP	AMC	CIP	FEP	CTX	CAZ	CXM
1	15	14	19	6	22	20	21	24	12	19	13	6	6	6
2	14	15	18	6	20	18	17	19	11	16	10	6	6	6
3	7	11	10	6	13	6	8	6	6	10	6	6	6	6
4	15	15	19	6	23	19	17	22	11	19	10	6	6	6
5	14	14	15	6	19	17	15	21	11	14	8	6	6	6
6	17	14	18	6	21	15	19	25	14	20	16	9	6	6
7	11	13	15	6	17	6	6	6	9	6	9	6	6	6
8	14	15	18	26	6	6	6	6	10	6	11	6	6	6
9	7	7	7	6	6	6	6	6	6	6	6	6	6	6
10	12	14	17	6	6	6	6	6	8	6	9	6	6	6
11	10	12	14	6	6	10	6	6	6	9	6	6	6	6
12	12	16	17	6	6	6	6	6	6	6	6	6	6	6
13	14	14	17	6	6	6	6	6	6	6	10	6	6	6

ETP, ertapenem; IMP, imipenem; MEM, meropenem; SXT, trimethoprim–sulfamethoxazole; AK, amikacin; CN, gentamicin; NET, netilmycin; TZP, piperacillin–tazobactam; AMC, amoxicillin–clavulanate; CIP, ciprofloxacin; FEP, cefepime; CTX, cefotaxime; CAZ, ceftazidime; CXM, cefuroxime.

Thirteen CRKP isolates were subjected to conventional PCR testing for the detection of common carbapenemase genes as described earlier. PCR amplified two genes: *bla*_NDM1_, 7 (2.18%); and *bla*_IMP4_, 6 (1.87%). PCR products were sent for DNA sequencing and compared with existing databases for confirmation. Gene accession numbers for *bla*_NDM1_ and *bla*_IMP4_ are listed in Table 2. A summary of phenotypic and genotypic results is given in Table 3.

**Table 2:** List of GeneBank accession numbers for *bla*_NDM1_ and *bla*_IMP4_ reference

Beta lactamase genes	GenBank Accession No.
*bla*_NDM1_	KC539432.1; KC539430.1; KC310727.1; JF798499.1; KF016990.1; AP012055.1; JN157804.1
*bla*_IMP4_	JX517203.1; KF250428.1; KF184388.1; JX457479.1; JN106667.1; AJ609296.3; FJ384365.1

## Discussion

This study provides the first reported data on the prevalence of CRKP among clinical isolates of *K. pneumoniae* in Kelantan, Malaysia. The prevalence of CRKP worldwide varies, partially depending on the cultural or population exchange relationship between countries and possible reservoirs of the carbapenemase producer (20). The prevalence of CRKP among *K. pneumoniae* was high in some regions: 13% in Greece, 8% in USA, 5.5% in Israel, and 5% in Argentina (4, 21–23).

**Table 3:** Summary of phenotypic and genotypic characteristics of carbapenem-resistant *K. pneumoniae*

	MIC (µg/ml) of carbapenem					
					
Isolate	IPM	MEM	MHT	CD-APB	CD-EDTA	Gene detected
1	0.5	2	+	–	+	*bla*_IMP4_
2	4	>32	+	–	+	*bla*_IMP4_
3	>32	>32	+	–	+	*bla*_IMP4_
4	8	>32	+	–	+	*bla*_IMP4_
5	4	>32	+	–	+	*bla*_IMP4_
6	0.25	2	+	–	+	*bla*_IMP4_
7	>32	>32	+	–	+	*bla*_NDM1_
8	>32	>32	+	–	+	*bla*_NDM1_
9	>32	>32	+	–	+	*bla*_NDM1_
10	16	8	+	–	+	*bla*_NDM1_
11	>32	>32	+	–	+	*bla*_NDM1_
12	16	8	+	–	+	*bla*_NDM1_
13	>32	>32	+	–	+	*bla*_NDM1_

MIC, minimal inhibitory concentration; IPM, imipenem; MEM, meropenem; MHT, modified Hodge test; CD-APB, combined disc with 3′ aminophenylboronic acid; CD-EDTA, combined disc with ethylene-diaminetetra-acetic acid.

More recently, some cases of CRKP were detected in neighboring countries. For example, in Singapore the OXA-181 genotype was found to be the second most common after NDM-1 producers (24). Vietnam detected their first two cases of CRE in 2010 (25). The isolated CRE were *Escherichia coli* and *K. pneumoniae* of NDM genotype. There is a possible danger that CRE and CRKP have emerged in Malaysia, as a result of the movements of individuals unknowingly or knowingly carrying CRE or CRKP.

In this study, the proportion of CRKP (4.05%) detected was lower than those previously reported elsewhere. Concomitant with this finding, studies in Singapore and Taiwan divulged that the prevalence of CRKP was <1 and 1.2%, respectively (26). In addition, only 1.17% was found in King Aziz Medical City, Riyadh, Saudi Arabia (10). These findings indicate that although the problems do exist, the prevalence of CRKP is still low in the Asian region (7).

The majority of the study on CRE focused on patients admitted in critical care and long-term care facilities with a higher rate of antibiotic exposure (27). In contrast, our study was not focused on any specific ward, but rather on a specific organism from various departments. Thus, being non-selective of patient population might have contributed to the lower prevalence of CRKP in our study. However, looking at the isolates distribution, the majority (61.5%) of CRKP were isolated from patients admitted to the ICU.

From sequencing analysis, only two genes, *bla*_IMP4_ and *bla*_NDM1_, were detected. These carbapenemase genes were increasingly isolated at rapid velocity. A study in Cipto Mangunkusumo Hospital in 2011 reported that 5% of CRKP were either *bla*_IMP_ or *bla*_NDM_ producers (28). In Malaysia, the first documented carbapenem-resistant case was *bla*_NDM_ in 2010 and the distribution was as low as <0.2% (29). Other carbapenemase genes were not discovered in our study as *bla*_SME_ and *bla*_IMI_ were chromosomally encoded carbapenemase. Being chromosomally encoded enzymes, with no evidence of mobile element association, they have not disseminated well globally. This fact may have contributed to their rarity and limited distribution (14), which subsequently suggests a smaller likelihood of it going viral in Malaysia.

The limitation of our study was that we did not screen other mechanisms related to high MICs shown in some of our isolates and the genetic relatedness among positive isolates. More clinical samples from hospitals with diverse bacterial species are required in order to determine the prevalence of carbapenem-resistant bacteria in Malaysia. In this manner a better understanding of CRE and CRKP may be achieved.

## Conclusions

In conclusion, the present study indicated that the prevalence of CRKP in our hospital setting was low. The data presented showed *bla*_NDM1_ and *bla*_IMP4_ were mainly responsible for the carbapenem resistance in our *K. pneumoniae* isolates. Further studies are warranted to determine other resistant mechanisms and also the genetic relatedness among these isolates.
